# The impact of tibial torsion measurements on gait analysis kinematics

**DOI:** 10.1590/1413-78522014220500579

**Published:** 2014

**Authors:** Paulo Roberto Garcia Lucareli, Nadia Maria Santos, Wagner De Godoy, Milena Moreira Barreto Bernal, Ângela Tavares Paes, Amancio Ramalho

**Affiliations:** 1 Universidade Nove de Julho, São Paulo, SP, Brazil, Universidade Nove de Julho, São Paulo, SP, Brazil; 2 Universidade de São Paulo, São Paulo, SP, Brazil, Universidade de São Paulo, São Paulo, SP, Brazil; 3 Hospital Israelita Albert Einstein, São Paulo, SP, Brazil, Hospital Israelita Albert Einstein, São Paulo, SP, Brazil; 4 Universidade Federal de São Paulo, São Paulo, SP, Brazil, Universidade Federal de São Paulo, São Paulo, SP, Brazil

**Keywords:** Tibia, Torsion abnormality, Biomechanical phenomena

## Abstract

**Objective:**

To measure and compare tibial torsion values as assessed by goniometry and three-dimensional kinematics. In addition, the impact of each one of these measurements on kinematic and kinetic results for normal gait was determined.

**Methods:**

Twenty-three healthy and fully ambulatory patients were assessed, 11 women and 12 men, from 20 to 40 years old. Data were collected at a laboratory for the three-dimensional analysis of movement with 10 cameras and two force plates. Tibial torsion measurements were obtained using goniometry and three-dimensional kinematics based on the Plug-in Gait model. Afterwards, both procedures were compared, and the impact of each result was assessed on the kinematic and kinetic modeling of the knee and ankle.

**Results:**

Pearson's linear correlation coefficient (r=0,504) showed a moderate correlation between the three-dimensional kinematics and goniometry, and between the changes in the measurements. Regarding the processed kinematic and kinetic results for every torsion position, no significant differences were noticed among any of the studied variables (p>0.05).

**Conclusion:**

Although statistical correlation among tibial torsion angles by goniometry and three-dimensional kinematic were moderate, kinematic and kinetic analysis of the joints did not reveal any significant changes. *Level of Evidence I, Diagnostic Studies - Investigating a Diagnostic Test.*

## INTRODUCTION

Three-dimensional gait analysis is an important tool to quantify and analyze standards of human locomotion under normal conditions or during disease states. However, accurate data regarding gait analysis may be seriously affected for the following reasons: for example, biomechanical model simplification, abnormality of soft tissues, deviations in the directions and position of joint centers and axes, and mistakes in anthropometrics measurement. Therefore, tibial torsion constitutes a relevant variable for assessing the spatial orientation of the tibia and ankle joint center, which are derived directly from this measure.

Tibial torsion has been defined in Helen Hayes's biomechanical model^1^^,^ 2 and in mainly three-dimensional gait analysis systems as the angle formed by the flexion-extension axis projection of the knee and the plantar flexion-dorsiflexion axis of the ankle in the transverse plane.

A number of methods have been proposed to measure tibial torsion,^3^^-^ 13 although common procedures used in gait analysis are clinical measurements using a goniometer between the transmalleolar axis (TMA) of the ankle and the longitudinal axis of the thigh as well as kinematics methods (referred to as gait analysis). This procedure determines the directions of the flexion-extension axis of the knee and plantar flexion-dorsiflexion of the ankle.^14^

This study aimed to measure and compare the degree of tibial torsion using goniometry (GTT) and three-dimensional kinematics (KTT) and to verify the impacts of both measurements on kinematic and kinetic results during gait analysis of normal individuals.

## PATIENTS AND METHODS

Twenty-three individuals (46 lower limbs) of both genders (eleven women and twelve men) between 20 to 40 years of age were selected with an average age of 26.2 ± 5.3 years. Participants were registered in the normals database of our laboratory and were collected. This study was approved by Albert Einstein Hospital ethics committee (726-09).

To participate in the study, individuals must have presented with community ambulation, without musculoskeletal impairments that could have affected ambulation, no pain complaint during gait and no history of a surgical procedure in the lower limbs at least six months before examination. Participants with a previous history of neuromuscular disorders such as seizures, tumors, heterotopic ossification, cognitive deficits and hearing and visual impairments were excluded.

**Figure 1:**
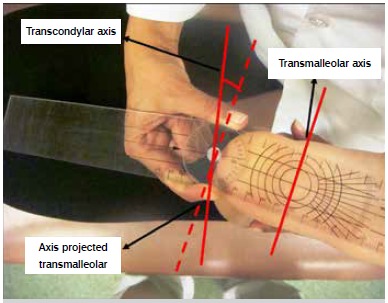
Transverse plane of the procedure for measuring tibial torsion with goniometry.

The GTT measurements were performed by two physiotherapists with more than five years of experience in gait analysis. Individuals were asked to lie in a prone position with their thigh extended and leg flexed at 90°. The goniometer fixed arm was defined on an imaginary line at the TMA using the medial and lateral malleolias as a reference. The mobile arm was placed on the longitudinal axis of the thigh assuming that this reference was the transcondylar axis.^15^ (Figure 1)

**Figure 2:**
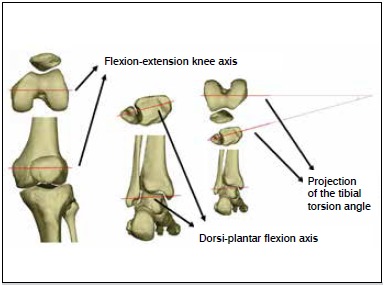
The tibial torsion angle formed by the projection of the axes of the plantar flexion-dorsiflexion of the ankle and the flexion-extension of the knee in the transverse plane of the tibia.

Using a Knee Alignment Device (KAD),^16^ a static trial was performed, and two more additional makers were placed at the medial malleoli to establish the plantar flexion-dorsiflexion axis of the ankle orientation. (Figure 2)

After the trials capture, the flexion-extension axis of the knee direction was recalculated to reduce the magnitude of valgus-varus motion during balance within the gait cycle, which was recommended by Baker et al.,^17^ KTT was measured between these two axes according to the model of Helen Hayes.^1^^,^ 2

Based on Helen Hayes's biomechanical model applied in Plug-in Gait(r) (PiG), markers were fixed on the volunteer's skin at pre-defined anatomical points to produce segments incorporating the pelvis, thigh, legs and feet.^1^^,^ 2 Three-dimensional data collection was performed using the VICON^(r)^ motion capture system, which utilized 10 cameras (MX-F40 model) and two AMTI^(r)^ force platforms. For processing and three-dimensional reconstruction, Vicon Nexus software (Oxford Metrics Group) was used.

For data analysis, eight gait cycle trials for each patient were processed twice. The first tibial torsion value was obtained using the clinical measurement and the second value was obtained during the kinematic static trial. A comparison was performed among the following thirteen characteristic points of the kinematic and kinetic graphics of the knee and ankle: angular position of knee on initial contact (KneeFlexExtIC); first peak of flexion of the knee during the support phase (KneeFlexExtLOAD); minimal angular value of the knee after peak of flexion during support

(KneeFlexExtMINAFP); angular position of the knee during pre-balance (KneeFlexExtFO); peak of flexion of the knee during balance (KneeFlexExtPKSW); angular position of ankle on initial contact (DorsiPlanFlexIC); maximum value of dorsiflexion during the support phase (DorsiPlanFlexPKD); minimal value for the plantar flexion position during the gait cycle (DorsiPlanFlexMIN); angular position of the ankle during pre-balance (DorsiPlanFlexFO); maximum value of internal moment for dorsiflexion during support (DorsiPlanFlexMMIN - Nm/Kg); maximum value of internal movement for plantar flexion during support (DorsiPlanFlexMMAX - Nm/Kg); maximum value of absorption potential during support (DorsiPlanFlexPMIN - Watts/Kg); and maximum value of potential produced during support (DorsiPlanFlexPMAX - Watts/Kg).

### Statistical procedure

To compare and measure methods for the tibial torsion calculation and the impact of these measurements on the kinematics and kinetics of gait, the Bland-Altman plot, Pearson's correlation coefficient and intraclass correlation coefficients (ICC)^18^ were used.

A p value less than 0.05 was considered statistically significant. For data analysis, the statistical programs SAS(r) (version 9.0) and SPSS(r) (version 17.0) were used.

## RESULTS

### Goniometry versus three-dimensional kinematics

**Figure 3:**
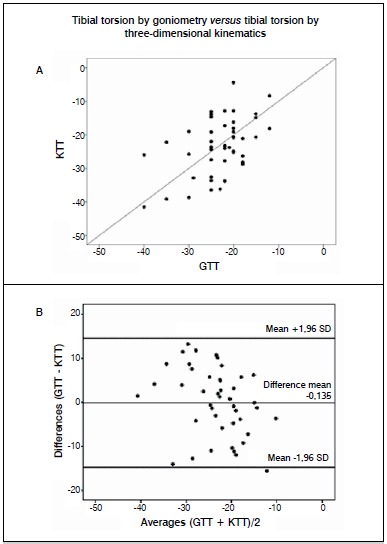
(A) Scatter plot showing the discrepancies between the absolute values of x TTG TTC. (B) Bland-Altman plot showing the balance between positive and negative differences.

Differences between GTT and KTT values were found to be -0.135, which was not statistically significant (p=0.903). Despite this finding, analyzing the measures individually (point to point) through dispersion graphs and a Bland-Altman plot, (Figure 3) revealed important differences changes. Using a signal test, there was an equilibrium between positive and negative differences (p=0.731). In other words, one method may not have underestimated or overestimated values of tibial torsion in relation to the other method.

Pearson's correlation linear coefficient (r) showed a correlation of 0.504 (p<0.001), but the intraclass coefficient correlation (ICC) indicated moderate concordance (ICC=0.488, confidence interval (CI) 95% = [0.230; 0.681]), thereby suggesting differences among these measures.

### Tibial torsion and three-dimensional analysis

Using the values for the tibial torsion obtained by these two methods, the aforementioned thirteen variables of the volunteers gait were analyzed (Table 1) to compare the kinematic and kinetic results. The both measures did not influence the values obtained for the studied variables, p> 0.05.

**Table 1:** Correlation and concordance between results for gait analysis obtained by two measurements of tibial torsion (goniometry and kinematics).

Variables	r	ICC	C.I. 95%
KneeFlexExtIC	0,99	0,989	[0,987; 0,991]
KneeFlexExtLOAD	0,996	0,996	[0,995; 0,996]
KneeFlexExtMINAFP	0,99	0,989	[0,987; 0,991]
KneeFlexExtFO	0,991	0,99	[0,989; 0,992]
KneeFlexExtPKSW	0,984	0,983	[0,980; 0,986]
DorsiPlanFlexIC	0,916	0,908	[0,892; 0,922]
DorsiPlanFlexPKD	0,995	0,995	[0,994; 0,996]
DorsiPlanFlexMIN	0,908	0,899	[0,882; 0,915]
DorsiPlanFlexFO	0,917	0,911	[0,895; 0,924]
DorsiPlanFlexMMIN	0,886	0,884	[0,861; 0,903]
DorsiPlanFlexMMAX	0,944	0,935	[0,922; 0,946]
DorsiPlanFlexPMIN	0,999	0,999	[0,999; 1,000]
DorsiPlanFlexPMAX	1	1	[1,000; 1,000]

## DISCUSSION

Measurement of tibial torsion, while important for the development of rehabilitation plans and for the structuring of accurate biomechanical models, has proven difficult as part of the procedures used in clinical gait analysis. The methods for measuring tibial torsion can be divided into clinical and kinematic aspects. This study used two methods to evaluate the statistical correlation between them and then measured the spread between their results regarding the kinetics and kinematics of the knee and ankle as determined by clinical gait analysis.

Comparing the results of torsion tibial measurements acquired by goniometry, TMA and three-dimensional kinematics of the knee and ankle showed a moderate correlation between these two techniques. However, observed changes were not predictive of one another.

Considering isolated values, the same techniques provided different results and the utilization of these values in biomechanical models demonstrated that there was no significant interference of the kinematic and kinetic data regarding the two joints.

There is a large amount of variation in tibial torsion values among individuals during goniometry testing by TMA. This variation may range from 0° to 45° in the external torsion angle of a normal population between 0 and 70 years of age^15^ with a medium angle of 20°. In volunteers, this variation was approximately 12° to 40°. It also was observed in the tibial torsion index; however, there were no significant differences among techniques.^8^

Goniometry was assessed by TMA and was compared with three other clinical techniques in cadavers, in vivo and in computer dissected cadavers. No correlation was demonstrated between the four clinical measures and direct measures of a lower recurrence rate in cadavers and *in vivo studies*, which was even lower when compared to other studies. These results may be subjectively supporteded using these four techniques, but the establishment of anatomic points was difficult, and there was a lack of accuracy in defining the longitudinal axis of the thigh. Additionally, the professional experience of the person who performed the test may lead to measurement differences.^19^

The technique's low repeatability was also noted when compared with methods of torsion marking by a paper footprint and with devices that more precisely establish the anatomic structures for the torsion calculations.^11^

Another study used a similar technique as described above to compare kinematic three-dimensional considerations with three other methods in order to estimate the axis of the knee. Herein, KAD values showed a correlation among the three three-dimensional methods, thereby suggesting that the physical examination was not an accurate measurement of tibial torsion.^14^

In relation to tibial torsion measures through ultrasonography, goniometry by TMA, the thigh-foot angle and the inclinometer exhibited good repeatability in the goniometry and ultrasonography assessments; however, only a weak to moderate correlation was observed.^12^

These results show that the degree of correlation between these tibial torsion techniques is not high, which may make it difficult to use two different measurement methods for the assessment of a patient.

The measurement of tibial torsion using clinical methods and a tendency to correlate the observed values from kinematic and kinetics data is commonly performed at gait analysis laboratories. Sometimes it is difficult to find concordance among these data, which is probably because the clinical measurement is static and the kinematic data are based on static positions that take movement into account.

Although both methods provide tibial torsion values, it is impossible to compare them or correlate their values or even replace one with the other. Thus, a relevant question to ask is if these clinical tibial torsion measurements are incorrect and are professionals that do not use tools for three-dimensional analysis performing procedures based on the wrong results?

Transcondylar angle, femoral neck and tibial torsion measurements when compared using three-dimensional computed tomography versus three-dimensional kinematic analysis of gait in normal children and children with cerebral palsy showed a low degree of correlation for normal children. However, for the children with cerebral palsy, the correlation was significant among measures of tibial torsion, pelvic and waist rotation, peak flexation of the knee upon first contact and hyperextension in pre-balance.^20^

Tibial torsion calculations using goniometry of the TMA with volunteers in prone and sitting positions and KAD comparing four distinct ways to define the movement of the knee axis determined the variables that affectgait of children with cerebral palsy. The authors noted that patients with malignment torsional defects may manifest changes in gait kinematics. For these patients, the obtained data did not truly represent the clinical alterations.^21^

Excessive external tibial torsion causes losses in foot stability and lift function in mid and terminal stance phases, thereby generating dysfunction in the muscle lever arm and restricting the efficiency of plantar flexion in ankle and knee stability during gait. This excessive torsionIt occurs upon the impact of the soleus and gastrocnemius muscles, thereby changing the action line and capability to produce muscle moment. Subsequently, this change affects the location and magnitude of force on the ground of the external movement and the body's three-dimensional dynamic.^22^^,^ 23

Correlations found among measurement techniques were based on studies that reached similar results. In addition, even those who did not performed a three-dimensional kinematic analysis showed poorer results than those observed in this study.^11^^,^ 12 ^,^ 14 ^,^ 19 ^,^ 20 However, when measures of tibial torsion through goniometry and three-dimensional kinematic were conducted using variables associated with kinematics and kinetics of gait, no impact was noted on the data analysis from healthy adult volunteers. This lack of impact might have been because computer models have shown that tibial torsion may cause kinematic and kinetic alterations in the sagittal plane of motion during gait.^22^^,^ 23 The biomechanical model applied was successful, and even when different tibial torsion values were introduced, there were no changes in the data. This fact also provides more confidence in the method of three-dimensional analysis. However, if there is no significant change in gait, then there remains a doubt of what method for determining tibial torsion calculations should be considered first. An excessive external or internal tibial torsion when presented requires special attention to determine its effect on biomechanical gait.

The most criticized possible bias associated with our study was the gait analysis reliability. A systematic review of the literature on inter-session and inter-assessor reliability showed that marker placement was the most likely source of error,^24^ which was also reported by Kadaba *et al*.^25^ To reduce that variance in our study, marker placement was performed by two experienced examiners in all cases. In the systematic review, the highest errors were found in transverse gait parameters for hip and knee rotation.^25^ However, these parameters were not examined in this study.

## CONCLUSION

Although clinical (GTT) and kinematic (KTT) measurements of tibial torsion vary significantly, there is very little effect on the resulting kinematic graphs of the sagittal plane for knee and ankle kinematics. We can further conclude that a comparison of most of the current methods reveals a wide degree of variability in tibial torsion. In this particular article, the fact that GTT and KTT varied significantly was not surprising given the inherent error of static measurements by a variety of technicians. In addition, the knee rotation was interposed between the thigh and the transcondylar axis duringkinematic measurement, and dynamic joint centering was apparently not performed. The fact that the sagittal plane of the knee and ankle motion were not significantly affected by wide variations in the inputted tibial torsion angle is probably the most salient point in this article.

ERRATA

The article entitled "ANALYSIS OF INJURIES' PREVALENCE IN SURFERS FROM PARANÁ SEACOAST" by Gabriela Chueiri de Moraes, Ana Tereza Bittencourt Guimarães, and Anna Raquel Silveira Gomes published on Acta Orthopedica Brasileira vol. 21 No. 04 -2013, pages 213-8, according to the authors' request, where it reads: **Level of Evidence II, Retrospective Study**, it shall read: **Level of Evidence III, Study of nonconsecutive patients; without consistently applied reference "gold" standard**.
